# Man and His Enemies

**Published:** 1889-06-08

**Authors:** 


### THE DOCTOR'S PULPIT.

### MAN AND HIS ENEMIES.

### FESTINA LENTE.

"LIFE is to be lived strenuously." When the oracle which gathers up the collective experience of modern times is honestly consulted, it steadily utters forth that. To the rightly constituted man, strenuousness is pleasure. Mr. Gladstone will not complain that his life has been one of manifold work done with all his might. Mr. W. G. Grace has probably found his keenest delights in his longest innings and his biggest scores. But there are opposite poles of strenuousness. There is the stolid, calm resistless weight of the elephant on the one hand, and, on the other, the yelping, cowardly fussiness of the lady's toy spaniel, who hates the cat, but dare not for his life go within a yard of her claws.

No man whose judgment is worth putting into words thinks Nature an unwise or unkind mistress because she gives him stern work to do. Most of those who stand out in sharp distinctness from their fellows, have an over-powering passion for work in some of its forms. These are the men who are best worth considering, alike from the moralist's, the statesman's, and the physiologist's point of view. The multitude are not to be despised; but he who would lead the multitude must address himself to its leaders. Leaders see the goal to be aimed at; but sometimes, in seeking the shortest road, they miss the quickest way. Mere speed may distance all competitors at the outset, but in a long race it often fails to come in first at the winning-post. The fable of the hare and the tortoise is more than two thousand years old. It will be two thousand more before all, even of clever men, will learn that it is not seldom the highest wisdom to "hasten slowly."

There are certain circumstances in life, and, on a more limited survey, certain periods of the year, which speak to a man bidding him "draw rein." At every decade a human being, who is at once both reasonable and mortal, may wisely remind himself that he is ten years older, and, therefore, ten years nearer the final failure of both bodily and mental powers. Has he concentrated the energy of twenty years on the operations of the last ten? If he has, it is pretty certain that he has expended all his stored resources of physiological capital, and has, moreover, borrowed extensively on the security of his future health. The expenditure of all the stored energy was foolish if it was not imperative. The borrowing on the security of future health was much more than foolish, and it may prove to have been ruinous. Such security with all men is most uncertain; with many it is absolutely worthless. There are ways and means of increasing "health capital," as there are ways and means of increasing other kinds of capital. And it is with health capital as with capital of all other kinds. He who has most of it, other things being equal, will make the largest number of successful and profitable transactions. If a man be really both strong-minded and practical, he will periodically take these questions of bodily and mental expenditure into consideration; and if he sees clearly that he has been making the pace too much, he will, at all costs, lower his fires, shut off steam, and slacken speed.

The summer season disposes to a certain feeling of restfulness. This is especially the case in the country. In the morning, the calm sunshine bathing the green fields and the trees in its beautiful light, makes a picture which few men can look upon without pleasure deep and lasting. The picture in its sweetness and calm is both a contrast and a protest against many of the ambitions, discontents, and ridiculous little worries with which a man allows his mind to be filled from day to day. "What is the value of mere wealth or high worldly distinction," it seems to say, "compared with the content and the simplicity which here prevail over all things, and which steal into the human mind by eyes and ears and every other sense? Here and now, merely to live is to be happy." Such a feeling, arising from such a cause, is Nature's gentle but monitory voice asking man to be reasonable, to be content, to give his worried brain a little rest, to let the hungry dogs of greed and ambition lie down and sleep.

It is staying power that wins the long race. But why should a man be everlastingly thinking about winning? Is there nothing in life and the world worth anything except conflict and victory? One gets sick of "the sound of the trumpet and the shouts of those that conquer." Pleasure—innocent pleasure; effortless movement; complete indifference to the conflict of life and everybody engaged in it; small amusements—such as cricket with the boys, or even "tipcat"; days well spent in utter idleness; early to bed, and late to rise; these are some of the occasional commandments of the modern gospel of science and health. The unthinking regard such commandments as entirely superfluous, being under the impression that all the world is too ready to forsake work and rush to play on the slightest encouragement. No greater mistake could be made. The unworthy majority may work because they are compelled, and for no other reason. But the few who really constitute the flower and perfect fruit of human kind often need the sharpest measures that can be devised to check them in their career of self-destruction from voluntary self-exhaustion. "Art is long, and life is short;" "whatsoever thy hand findeth to do, do it with thy might;" "work whilst it is called day for the night cometh,"—these and the like are wise and right when wisely and rightly used. But they can be, and often are, abused by men of the best motive and the purest life. The other side of the question, too, must be looked at; and as Nature has placed limits to our powers, so we must place limits to our work. The eleventh commandment added to every eager and resolute man's decalogue may properly be "Festina Lente."

The medicine of rest, of leisure, of a mind in repose, is but little valued by the multitude. Yet what physician of any experience does not know, not only of its inestimable worth, but also that in many cases it is the one thing of all others which is of real use to the patient? There is not a drug known to science which can even temporarily act as an efficient substitute for quietness of mind. To be in perpetual excitement and to hope to allay its evil effects by the physic bottle is the practice of a madman. He who works hard must rest quietly and sleep soundly.

